# Characterization of Speech and Language Phenotype in GLUT1DS

**DOI:** 10.3390/children8050344

**Published:** 2021-04-27

**Authors:** Martina Paola Zanaboni, Ludovica Pasca, Barbara Valeria Villa, Antonella Faggio, Serena Grumi, Livio Provenzi, Costanza Varesio, Valentina De Giorgis

**Affiliations:** 1 Department of Child Neurology and Psychiatry, IRCCS Mondino Foundation, 27100 Pavia, Italy; martinapaola.zanaboni@mondino.it (M.P.Z.); barbaravaleria.villa02@universitadipavia.it (B.V.V.); antonella.faggio@mondino.it (A.F.); serena.grumi@mondino.it (S.G.); livio.provenzi@mondino.it (L.P.); costanza.varesio@mondino.it (C.V.); valentina.degiorgis@mondino.it (V.D.G.); 2 Department of Brain and Behaviour Neuroscience, University of Pavia, 27100 Pavia, Italy

**Keywords:** GLUT 1 transporter deficiency syndrome (GLUT1DS), language, speech, oral motor, dysarthria

## Abstract

Background: To analyze the oral motor, speech and language phenotype in a sample of pediatric patients with GLUT 1 transporter deficiency syndrome (GLUT1DS). Methods: eight Italian-speaking children with GLUT1DS (aged 4.6–15.4 years) in stable treatment with ketogenic diet from a variable time underwent a specific and standardized speech and language assessment battery. Results: All patients showed deficits with different degrees of impairment in multiple speech and language areas. In particular, orofacial praxis, parallel and total movements were the most impaired in the oromotor domain; in the speech domain patients obtained a poor performance in the diadochokinesis rate and in the repetition of words that resulted as severely deficient in seven out of eight patients; in the language domain the most affected abilities were semantic/phonological fluency and receptive grammar. Conclusions: GLUT1DS is associated to different levels of speech and language impairment, which should guide diagnostic and therapeutic intervention. Larger population data are needed to identify more precisely a speech and language profile in GLUT1DS patients.

## 1. Introduction

GLUT 1 transporter deficiency syndrome (GLUT1DS) is a rare, treatable, metabolic encephalopathy due to mutations in SLC2A1 gene [1], which causes a non-functional glucose uptake by GLUT1 transporter, primarily expressed in endothelial cells forming the blood-brain barrier and in astrocytes [2]. Ketogenic dietary therapies (KDTs) are recognized as the gold standard treatment for GLUT1DS since they provide alternative fuel, namely ketone bodies, for brain energy metabolism [2]. Symptoms develop in age-specific pattern [2] and the classical disease phenotype includes a wide range of movement disorders, drug-resistant epilepsy, neurodevelopmental impairment, and acquired microcephaly. Moreover, ataxia, dystonia, dysarthria, persistent tremor, spasticity are typical findings at neurological examination. Milder and atypical phenotypic variants are continuously reported and GLUT1DS phenotypic spectrum is progressively expanding. KDTs introduction, especially when occurring early in life, could lead to improvement of certain symptoms, as often occurs with epileptic manifestations, intellectual and social adaptive skills [3], whereas its beneficial effects on movement disorders are less evident [2].

Intellectual disability (ID) is a usual finding in GLUT1DS patients, ranging from severe to mild; only a minority of affected patients shows normal intelligence quotient (IQ) [4]. Genotype-phenotype correlation has not been clearly defined so far. Some reports described a milder phenotype or later disease onset in patients with missense mutations or higher cerebrospinal fluid (CSF)/blood glucose ratio [5]. The existence of a proportional relationship between ID and disease severity is debated: there have been reports both supporting and disproving this relation [6,7,8]. De Giorgis et al. defined a typical cognitive phenotype of GLUT1DS [4], evaluating the neuropsychological profile of 25 patients before and after KDTs introduction. The typical phenotype observed was characterized by a performance IQ more affected than verbal IQ (VIQ), together with greater difficulties in visuospatial and visuomotor skills. In the same study, a significant direct correlation between IQ (total IQ (TIQ) and VIQ) and CSF/blood glucose ratio values was observed.

Speech and language functions in patients with GLUT1DS are the least documented in literature and, to the best of our knowledge, they have only been assessed in the context of broad neuropsychological batteries [5,6,9,10] and have not been deeply characterized yet. To date, the presence of different degrees of speech and language impairments, with varying degrees of motor incoordination, have been described as common features in children and adults with GLUT1DS [2]. In particular, Hully and colleagues [6] reported language delay with dysarthric speech in almost 80% of GLUT1DS patients. In the study by Ramm-Pettersen et al. [10], an improvement of those aspects was recorded after KDTs introduction: the caregivers of six patients reported progress concerning general alertness, expressive language, articulation, and physical endurance in the wake of the dietary treatment. The sooner the KDTs is introduced, the greater is its potential of changing the disease course [2].

The phenotypic variability and the different response to KDTs therapy according to introduction timing should be read considering that glucose utilization in the brain increases threefold from infancy to 3 years of age [11] and thus, for patients with GLUT1DS, that period represents a critical frame if left untreated. Moreover, the neuroconstructivism approach underlies the bidirectional interactions between human biology and social environment, the development itself might be seen as playing a crucial role in shaping phenotypical outcomes [12].

We believe that recognition of a typical speech and language profile might be crucial for disease assessment and targeted rehabilitation.

For this purpose, the present study aimed to characterize in detail language development and to analyze the oral-motor, speech and language phenotype in a pediatric population of patients with GLUT1DS, through the use of standardized tests.

## 2. Materials and Methods

### 2.1. Participants

This was a mono-center study. Among 25 patients with established clinical and genetic diagnosis of GLUT1DS of all ages treated with KDTs and regularly followed at our clinic [4], we included eight monolingual Italian-speaking patients (four females and four males), aged 4.6–15.4 years (median age 10; standard deviation (SD) 3.79; range 4.6–15.4 years).

For each patient, information such as sociodemographic and clinical variables were collected (child’s pre-peri and post-natal clinically events, developmental history, presence of otitis’ history or auditory impairment, previous diagnosis other than GLUT1DS, time of GLUT1DS diagnosis, CSF/blood glucose ratio, SLC2A1 mutation, family history of GLUT1DS, family history of epilepsy, epilepsy history, presence of movement disorder, KDTs and rehabilitation initiation timing) (see Table 1).

In detail, five patients have a missense mutation, two patients have a deletion of SLC2A1 gene and one patient a truncating mutation. Pregnancy was uncomplicated in all but one patients. No patient suffered from recurrent otitis media during the first years of life, nor auditory deficits. Based on parental reports, the following information was obtained: babbling onset was mainly delayed (age 13.29 months; SD 5.61; range 9–24) and so was the age of first-word onset (age 18.25 months; SD 7.2; range 12–30) and age of combinatory speech (age 34.5 months; SD 16.27; range 24–60). Three patients showed unintelligible speech during the preschool years. Psychomotor development was delayed in two patients. Before the genetic and clinical diagnosis of GLUT1DS, three patients were diagnosed with a speech disorder and two patients with ‘dysarthria and ataxia’ of unknown origin at first disease manifestations. Age at GLUT1DS diagnosis ranged from 24 to 122 months (mean 72.87 months; SD 26.70). Two patients had a family member affected by GLUT1DS and four patients had a family history of epilepsy. Epilepsy onset in the described sample occurred from 12 to 84 months (mean 36.28 months; SD 24.71). The median age at KDTs initiation was 76.75 months (SD 29.85; range 24–121 months). All patients had movement disorders: seven with mild severity and one with moderate severity. Rehabilitation was provided in all cases but one: six patients underwent speech and language therapy, four patients psychomotor therapy, and one patient underwent cognitive rehabilitation. Rehabilitation started at a median age of 49.71 months (SD 30.53, range 24–108) and the therapy duration reported lasted from 1.5 years to eight years (median 4.78 years, SD 2.57).

All patients were on stable KDTs therapy from more than six months at the evaluation time.

### 2.2. Materials and Procedures

All patients underwent a cognitive and speech and language evaluation. Speech and language assessment investigated three domains: oromotor, speech, and language abilities (see Table 2 for a detailed list of standardized tests performed).

Test administration was carried out individually by a professional neuropsychologist and a speech and language therapist. Both speech and language and cognitive assessments were performed by administering a comprehensive battery of tests depending on patient’s age. Language development patterns were reviewed by an expert neuropsychologist and a speech and language therapist through a standardized questionnaire and by collecting detailed medical and developmental milestones history.

This study was approved by our Ethical Committee (P-20190033749), IRCCS Mondino Foundation, Pavia. Written informed consent was obtained from caregivers.

### 2.3. Statistical Analysis

We firstly performed a set of descriptive analyses. Then, a set of Spearman’s rank correlation (rho) coefficients were calculated between the age onset of babbling, age at first word, age at combinatory speech, and the neuropsychological tasks’ results.

## 3. Results

### 3.1. Tests Evaluation

#### 3.1.1. Oromotor Skills

Oromotor skills were evaluated with nonverbal tasks, and demonstrated impaired functioning on at least one subtest. The worst performances were obtained in orofacial praxis verbal requests, parallel movements, and total score. In particular, in orofacial praxis verbal request (mean z-score −2.72, SD 3.1; range −9.66 0.75) 4/8 subjects showed a severely impaired performance and 3/8 had a poor performance; in ‘parallel movements’ 5/8 subjects scored a severely impaired performance (mean z-score −3.08, SD 3.42; range −6.09–1.10) and in ‘total score verbal request condition’ (mean z-score −2.63, SD 2.29; range −6.83 0.43) 4/8 patients had a severely impaired result and 2/8 patients a mildly impaired score. See Table 3.

#### 3.1.2. Speech

All but one individual demonstrated impaired articulation, characterized by imprecise production of consonants (7/8) and vowels (1/8). Only one child had acquired all speech sounds expected for his age. Most absent consonants were vibrant [r] (7/8), followed by voiced palatal lateral approximant [ʎ] (6/8, fricatives [z], [s] (2/8) and affricates [dz], [ts] (2/8), both voiced and voiceless. Phonological planning was strongly impaired in all children but one, who showed a mild impairment (mean z-score −15.24, SD 9.52; range −31.5 −1.29). All patients showed a high percentage of phonetically inaccurate production of words.

We identified simplification processes (backing 3/8; fronting 4/8; stopping 3/8; epenthesis 6/8; metathesis 2/8; cluster reduction 5/8; gliding 1/8; voicing2/8; de-voicing 3/8; affrication 2/8; affrication 3/8; assimilation 4/8; diphthong reduction 2/8) and atypical processes (stops deletion/reduction in clusters 3/8; conflicting processes 3/8; idiosyncratic processes 4/8). Speech was typified by the imprecise articulation of consonants and vowels, abnormal nasal resonance, low pitch, and prosodic errors (e.g., excessive stress on unstressed parts of speech, slow rate, short phrases). Evaluation of Diadochokinesis rate was significantly slower in 3/7 patients, mildly impaired in 1/7 (mean z-score 0.7, SD 3.48; range −7.86 0.8). Conversation speech intelligibility was adequate in seven out of eight patients. See Table 3.

#### 3.1.3. Language

Lexical abilities were preserved in 6/8 patients, both in receptive (mean standard score 89.63, SD 13.42; range 75–113) and expressive vocabulary (mean z-score −0.91, SD 2.01; range −5.45 0.77). Receptive vocabulary resulted in the normal borderline range in 2/8 patients. Expressive vocabulary was severely impaired in one patient, moderately impaired in two patients, and normal in five patients. Receptive grammar was the most impaired domain (mean scaled score 4.75, SD 2.81; range 1–8): a severe impairment was seen in 4/8 patients, two patients showed a borderline normal score, and 2/8 patients obtained a normal score. Expressive grammar (mean scale score 7.37, SD 3.42; range 1–12) was found impaired just in 1/8 patients, 3/8 performed borderline normal and 4/10 normal. See Table 3.

#### 3.1.4. Intelligence Quotient

Full-scale IQ (FSIQ) scores varied from moderately impaired to normal (range 41–96; median 73.38; SD 15.55). Verbal Comprehension Index (VCI) was more conserved compared to the remaining sub-IQ, with a median score of 85 (SD 11.11; range 66–98). See Table 3.

### 3.2. Correlations

The results of the Spearman correlation indicated that there was a significant negative association between age at the babbling onset and phonemic fluency tasks’ score and phonemic inventory. Age at the babbling onset was inversely related to phonemic fluency task and the number of consonants acquired: the greater the delay in babbling’s onset, the smaller the number of words produced at the phonemic fluency task [ρ(5) = −0.89, *p* = 0.042] and the number of consonants acquired [ρ(7) = −0.90, *p* = 0.005]. Age at the first word onset significantly correlated with phonemic fluency tasks’ score [ρ(5) = −0.91, *p* = 0.03] and phonemic inventory [ρ(8) = −0.71, *p* = 0.049]. Children who produced their first word earlier had a better performance in phonemic fluency tasks [ρ(5) = −0.91, *p* = 0.03] and they had a greater number of stable consonants [ρ(8) = −0.71, *p* = 0.049]. See Table 3.

Moreover, we attempted a correlation between the severity of language evaluations (lower scores in language and oromotor assessments) and genotype. Due to the small sample, we could not find a significance; in particular, as reported in literature about clinical phenotype severity [7], patients with deletions (*n* = 2) or truncating mutation (*n* = 1) do not seem to have a more impaired profile compared to patients with missense mutations (*n* = 5).

The same correlation was attempted between language evaluation and CSF/serum glucose ratio without significative results due to the small numbers of patients (*n* = 6) and the small range of values (mean 0.35, range 0.27 −0.043).

It has not been feasible to search for different functioning trajectories according to age of KDTs initiation. Nevertheless, in our sample, patients who started KDTs after six years of age achieved lower scores in language assessment (*n* = 4, mean 100.25 months, range 88–121 months). Regarding oromotor skills, we did not observe the same trend.

## 4. Discussion

Speech and language impairment have already been recognized in patients with GLUT1DS, but have not been fully characterized compared to other disease symptoms. In available studies, language functioning in GLUT1DS is depicted as extremely variable, ranging from no apparent deficit to the absence of expressive speech, with most affected individuals having reduced language skills [22,23]. In the present study, we deeply investigated speech and language profile in eight Italian-speaking children with GLUT1DS.

Based on parental reports, we documented a delay of early vocal behavior and early language milestones with a late onset of first word and combinatory speech in the majority of patients. We also found a significant negative association between babbling onset and the number of words produced in the phonemic fluency task and phonetic inventory. The delay in the mean age of babbling onset represents a crucial finding, since several studies support the predictive value of babbling onset timing and characteristics to determine subsequent speech and language abilities and communication disorders [24,25]. Babbling represents a linguistic and articulatory exercise and the experience of frequent self-producing consonants and vowels syllables makes infants more aware of similar patterns in their environmental language, acting as potential building blocks for word representations [25]. Moreover, in our sample the age at the first word onset significantly correlated with phonemic fluency tasks’ score and phonemic inventory, meaning that children who produced their first word earlier had a better performance in phonemic fluency tasks and a greater number of stable consonants. Importantly, it is often hypothesized that the first speech-like articulation and the babbling phase, which occur at approximately ten months of age, allow infants to develop a link between articulatory settings and the resulting auditory consequences, thus contributing to the development of the phonetic inventory and adaptation to the ambient language [26]. In this connection, the early signs of speech and language deviance and slow acquisition of expressive words in the second and third years of life may set off a cascade, negatively affecting a variety of following additional linguistic capabilities [24]. This scenario, which is frequently reported in cognitive and language disorders, has never been described as associated with GLUT1DS previously.

Oral-motor skills were impaired in most subjects in our sample. Development of orofacial praxis is impaired in a series of developmental disorders such as Developmental Coordination Disorder, Developmental Apraxia of Speech and Speech disorder [27]. These conditions have in common the combined presence of motor and language deficits, as observed in patients with GLUT1DS.

Speech was often characterized by phonetically inaccurate production of words, imprecise articulation of consonants and vowels, abnormal nasal resonance, low pitch, and prosodic errors. The most represented impairment was found in the phonological planning. This task resulted as severely deficient in seven out of eight patients, confirming the presence of a speech and language disorder, still active in some patients, and partially compensated in others. Receptive and expressive language abilities revealed different degrees of impairment in our patients; some of them showed severe receptive and expressive linguistic deficits, others had a mild impairment and only one had a normal profile. In all patients, a more conserved expressive and receptive lexical competence was observed, while linguistic grammar ability was impaired with a greater compromise of the receptive abilities. We may assume that a severe impairment at the morpho-syntactic level of language organization could be interpreted as the less likely domain to recover in patients with a previous speech and language disorder, as observed in GLUT1DS patients.

Several reports describe a mild-to-severe intellectual disability of GLUT1DS patients, in most cases proportional to the disease’s severity [6,7,8]. In our sample, FSIQ scores varied from moderately impaired to normal, one child showed a normal intelligence, five patients had a borderline intellectual functioning, two patients received a diagnosis of intellectual disability on mild and moderate ranges. VCI showed up as more conserved compared to the remaining sub-IQ: these data confirm the results of our previous work, where PRI was more affected than VCI [4]. A less impaired verbal quotient could lead at first to a misidentification of language deficits but, as shown by our results, an impairment of several linguistic domains can be documented with focused tests.

Due to the small number of patients included, it has not been feasible to obtain a phenotype-genotype and/or a phenotype/glycorrhachia correlation, as well as to search for different functioning trajectories according to age of KDTs initiation or total IQ level.

Nevertheless, in our sample, patients who started KDTs later in life (mean 8.5 years) achieved lower scores in language assessment and the patient with lowest IQ achieved one of the worst performances. Definitely larger samples are needed to assess whether KDTs initiation timing and mutation type might influence chances of recovery of speech and language. Unfortunately, in our sample KDTs introduction was late for all included patients.

Children with GLUT1DS are at a disadvantage in the development of cognitive functions since the disease itself causes a lower supply of energy for the correct functioning of the brain, resulting in a multilevel dysfunction affecting cognitive, speech and language abilities, as evidenced by the neuropsychological and language assessment carried in our sample [4]. Our data confirm the presence of a potentially heterogeneous cognitive and linguistic profile with different degrees of impairment in multiple speech and language areas. The variability of the linguistic profiles observed could be explained based on the general theoretical framework of neuroconstructivism [12].

This model is suitable to understand the interaction between biological and socio-environmental factors determining the linguistic development of patients with GLUT1DS.

The neuroconstructivism approach highlights how tiny variations in the initial state could give rise to domain-specific differences in end states [12]. If brain energy requirements are not satisfied in the first years of life, an impairment of input processing and starting points such as language circuitry will occur. Variability of genetic mutations, adaptive strategies, successful behavior as well as intact domains leads to inter-individual outcome differences, that could explain the relative heterogeneity of language profile in our small sample. We did not find factors determining language outcome; nevertheless, we believe that focus must be placed in at-risk populations in early infancy, even before onset of language, and that this time window should represents the optimal timing to start therapy, namely KDTs.

Limitations of this study are represented by the small number of subjects included—also due to the low prevalence of the disease—the age heterogeneity and the absence of a language and speech assessment before KDTs introduction.

## 5. Conclusions

In conclusion, GLUT1DS can be considered a multilevel condition affecting cognitive, motor, speech, and language competences. Our results confirm the importance of a complete speech and language evaluation to obtain a detailed profile, that is crucial to plan early and specific rehabilitative intervention.

GLUT1DS patients are often diagnosed with aspecific language disorder or delay in the first years of life, before other symptoms manifest. In this scenario, recognizing typical and atypical language fragilities and searching for a common linguistic phenotype in these patients could help to guide early diagnosis. An early diagnosis of GLUT1DS would allow a prompt start of target dietary treatment and of rehabilitative intervention inclusive of speech and language training. Further studies are needed to evaluate the effects of KDTs on language function.

## Figures and Tables

**Table 1:** Patients’ clinical history

	Pt1	Pt2	Pt3	Pt4	Pt5	Pt6	Pt7	Pt8	All (*n* = 8)
Age at evaluation (years)	6.1	13.9	15.4	4.6	10.3	11.2	11.4	7.11	M 10 (SD 3.79) range 4.6–15.4
Age at GLUT1DS Diagnosis (months)	59	60	75	24	122	79	95	69	M 72.87 (SD 26.70) range 24–122
CSF/serum glucose ratio	0.37	NA	0.34	0.27	NA	0.35	0.43	0.37	-
Genotype	DeletionExones1–10	Missense R400C	Deletion 1p34.2	Truncatin Y366X	Missense G314S	Missense N34S	Missense G17R	Frameshift L486fs	-
GLUT1DS Family history	no	no	no	no	yes	yes	no	no	yes 25%
Perinatal Period	un	un	un	un	un	un	un	Prematurity jaundice	yes 12.5%
Recurrent otitis	no	yes	no	no	no	no	no	no	yes 12.5%
Auditory deficits	no	no	no	no	no	no	no	no	yes 0%
Babbling onset (months)	18	12	24	10	9	10	10	na	M 13.29 (SD 5.61) range 9–24
Age at first word (months)	30	24	24	20	12	12	12	12	M 18.25 (SD 7.2) range 12–30
Age at combinatory speech (months)	60	24	60	24	24	24	24	36	M 34.5 (SD 16.27) range 24–60
Preschool speech intelligibility	no	yes	no	no	yes	yes	yes	yes	no 37.5%
Language disorder	dysarthria	speech	dysarthria	speech	speech	none	none	none	speech 37.5% dysarthria 25%
Psychomoto development	delayed	normal	delayed	normal	normal	normal	normal	normal	delayed 25%
Intellectual disability	borderline	borderline	moderate	normal	borderline	borderline	mild	borderline	-
Epilepsy Family history	yes	no	yes	no	yes	yes	no	no	yes 50%
Epilepsy	yes	yes	yes	no	yes	yes	yes	yes	yes 87.5%
Epilepsy onset (months)	24	36	26	na	84	60	12	12	M 36.28 (SD24.71) range 12–84
Movement disorder	yes	yes	yes	yes	yes	yes	yes	yes	yes 100%
Movement disorder severity	moderate	mild	mild	mild	mild	mild	mild	mild	mild 87.5% moderate 12.5%
Age at KDTs Initiation (months)	60	60	98	24	121	88	94	69	M 76.75 (SD 29.85) range 24–121
Rehabilitation therapy	speech	psychomotor & speech	speech	psychomotor & speech	speech & cognitive	psychomotor & speech	none	psychomotor	speech 75% psychomotor 50% cognitive 12.5%
Rehabilitation onset (months)	24	60	24	24	108	60	na	48	M 49.71 (SD 30.53) range 24–108
Rehabilitation’s duration (years)	6	6	8	3	1.5	7	na	2	M 4.78 (SD 2.57) range 1.5–8

Abbreviations: M, mean; SD, standard deviation; n, number; na, not available; KDTs, Ketogenic dietary therapies; Un, unremarkable.

**Table 2:** Description of the tests included in the administered battery.

Task	Description
Parental report on clinical history	Child’s pre-peri and post-natal clinically events and speech and language milestones acquisition.
Oromotor skills	Oromotor skills were examined with Orofacial Praxis [13]. Oromotor skills were the ability to plan and execute movements or sequences of voluntary movements, meaningful or not, using the muscles of the pharyngo-buccofacial system or the orofacial region. The Orofacial Praxis Test, consisting of 36 gestures, 24 single and 12 complex, elicited through verbal and imitative request.
Phonetic inventory	Phonetic inventory was investigated with the Articulation Test of Fanzago [14].This instrument was based on spontaneous/repetition elicited denomination of 114 figures which named allow to verify whether the target phoneme (place in different positions within the word) has been produced correctly or replaced/omitted/distorted.
Phonological Planning	Phonological Planning was tested by the Repetition of 31 words pronounced by the examiner [15]. This subtest is designed to assess phonological encoding and decoding through the repetition of words and it allows to detect the presence of phonological processes. For each word it is possible to calculate the number and the type of phonological processes produced. It is possible to identify two phonological processes, simplification and atypical. Simplification processes represent the persistence of normal primitive processes in successive stages of phonological development. Atypical idiosyncratic processes included types of simplifications rarely found in normal language development, or those that that are never found in normal developmental processes.
Diadochokinesis	Diadochokinesis was assessed with Maximum performance rate [16]. This task is used to test the ability to repeat a syllable sequence (/pataka/) as quickly as possible for 20 s in order to look at motor speech skills separate from the effects related to word familiarity.
Receptive vocabulary	Receptive vocabulary was evaluated by PPVT-III [17]. The PPVT is a receptive vocabulary test in which the child points to one of four pictures on a page that is named by the examiner.
Expressive vocabulary	Expressive vocabulary was tested by the Name BVN 5-11 [18]. For this task, the subject is asked to name 20 (for children aged from 5 to 11) or 88 (for children aged from 12 to 18) figures in order to measure patient’s vocabulary ability.
Receptive grammar	Receptive grammar was examined with Comprehension of Instructions NEPSY [19]. This task assesses receptive language and it involves understanding verbal instructions and processing them into actions.
Expressive grammar	Expressive grammar was evaluated by Sentence repetition NEPSY II [19]. This task was used to investigate the production of grammar structures.
Verbal Fluency	Verbal Fluency was examined with Word generation NEPSY II [19]. This subtest is designed to assess verbal productivity through the ability to generate words and it consists of two tasks: semantic or phonemic fluency. The participants are given 1 min to generate as many words as possible within a semantic category or they are asked to say words that start with a given letter.
Cognitive assessment	Age-appropriate versions of the Wechsler scales were administered to assess intellectual ability: —from 6 to 16 years the Wechsler Intelligence Scale for Children—Fourth Edition [20] or 2–6 year olds, the Wechsler Preschool and Primary Scale of Intelligence—Third Edition, Italian version (WPPSI-III) [21] and –Full-scale IQ (FSIQ) scores were derived and classified according to test manual normative data.

**Table 3:** Results of neuropsychological assessment.

	Pt1	Pt2	Pt3	Pt4	Pt5	Pt6	Pt7	Pt8	All (*n* = 8)
Voiced praxis verbal request (zs)	−2	1.37	−2	−1.70	−3.68	−1.16	1.37	−0.3	M −1.01 (SD 1.74) range −3.68 1.37
Voiced praxis imitation (zs)	−3.22	0.54	0.54	−1.17	−5.11	−1.33	0.54	−3.22	M −1.55 (SD 2.12) range −5.11 0.54
Orofacial praxis verbal request (zs)	−1.33	−3.41	−1.33	−2.11	−9.66	−3.41	0.75	−1.33	M −2.72 (SD 3.1) range −9.66 0.75
Orofacial praxis imitation (zs)	−1.90	0.53	−1.73	0.71	−1.90	−1.90	0.53	0.53	M −0.64 (SD1.3) range −1.9 0.71
Sequence movements verbal request (zs)	−2.91	−0.5	−2.84	0.38	−0.5	−0.5	0.69	−2.91	M −1.13 (SD 1.51) range −2.91 0.69
Sequence movements imitation (zs)	−4.74	0.43	−3	−0.28	0.43	0.43	0.43	−1.29	M −0.94 (SD 1.95) range −4.74 0.43
Parallel movements verbal request (zs)	−6.09	−2.80	0.69	1.10	−6.09	−6.09	−6.09	0.69	M −3.08 (SD 3.42) range −6.09 1.10
Parallel movements imitation (zs)	−10.31	0.21	0.21	0.75	0.21	−5.05	−5.05	0.21	M −2.35 (SD 4.03) range −10.31 0.75
Total score verbal request (zs)	−4.41	−0.77	−3.2	−1.15	−6.83	−3.2	0.43	−1.98	M −2.63 (SD 2.29) range −6.83 0.43
Total score imitation (zs)	−6.26	0.75	−1.87	0.74	−2.75	−1.87	−0.12	−1.52	M −1.61 (SD 2.27) range −6.26 0.75
Phonetic inventory: absent phonemes (des)	d g tʃ dȝ s z ts dz r ʎ	r ʎ	z s r ʎ st ts	r ʎ	none	r	ʎ r	ts dz ʎ r	-
Present phonemes (consonants and vowels) (*n*)	16	24	22	24	26	25	25	22	M 23 (SD 3.16) range 16–26
Phonological Planning (zs)	−31.5	−10.96	−22.38	−9.73	−21.61	−9.23	−1.29	na	M −15.24 (SD 9.52) range −31.5 −1.29
Phonological Planning (word accuracy) (*n*)	0/31	24/31	20/31	8/31	18/31	26/31	29/31	na	-
Phonological Planning (syllabic structure accuracy) (*n*)	26/31	28/31	28/31	31/31	30/31	31/31	31/31	na	-
Phonological Planning (word length accuracy) (*n*)	8/31	24/31	26/31	15/31	25/31	30/31	31/31	na	-
Diadochokinesis (zs)	−7.86	−0.53	−1.2	−3.2	−0.86	−3.2	0.8	na	M −0.27(SD 3.48) range −7.86 0.8
Receptive vocabulary (st)	75	113	83	106	91	87	85	77	M 89.63 (SD 13.42) range 75–113
Expressive vocabulary (zs)	−1.9	0.03	−5.45	−0.1	−1	0	0.35	0.77	M −0.91 (SD 2.01) range 5.45–0.77
Receptive Grammar (ss)	3	6	1	8	8	1	5	6	M 4.75 (SD2.81) range 1–8
Expressive Grammar (ss)	7	9	1	10	6	5	9	12	M 7.37 (SD 3.42) range 1–12
Word generation: semantic fluency (ss)	3	7	3	9	4	6	4	9	M 5.62 (SD 2.5) range 3–9
Word generation: phonemic fluency (ss)	na	3	3	na	6	6	4	na	M 4.40 (SD 1.51) range 3–6
FSIQ (st)	76	81	41	96	71	73	69	80	M 73.38 (SD 15.55) range 41–96
VCI (st)	88	82	66	98	90	84	74	98	M 85 (SD 11.11) range 66–98
PRI (st)	71	102	48	93	80	76	65	73	M 76 (SD 16.56) range 48–102
WMI (st)	na	76	52	na	64	76	91	na	M 71.80 (SD 14.63) range 52–91
PSI (st)	58	79	56	na	74	82	82	67	M 71.14 (SD 10.99) range 56–82

Abbreviations: zs = zeta score; M = mean; SD = standard deviation; des = descriptive; n = number; na = not available; ss = scaled score; FSIQ = Full scale intelligence quotient; st = standard score; VCI = Verbal Comprehension Index; PRI = Perceptual Reasoning Index; WMI = Working memory index; PSI = Processing speed index.
